# *Bacillus* spp. Inhibit *Edwardsiella tarda* Quorum-Sensing and Fish Infection

**DOI:** 10.3390/md19110602

**Published:** 2021-10-23

**Authors:** Rafaela A. Santos, Marta Monteiro, Fábio Rangel, Russell Jerusik, Maria J. Saavedra, António Paulo Carvalho, Aires Oliva-Teles, Cláudia R. Serra

**Affiliations:** 1 Departamento de Biologia, Faculdade de Ciências, Universidade do Porto, Rua do Campo Alegre s/n, Ed. FC4, 4169-007 Porto, Portugal; mailingmarta@gmail.com (M.M.); fjorangel@gmail.com (F.R.); apcarval@fc.up.pt (A.P.C.); 2 CIIMAR—Centro Interdisciplinar de Investigação Marinha e Ambiental, Terminal de Cruzeiros do Porto de Leixões, Av. General Norton de Matos s/n, 4450-208 Matosinhos, Portugal; saavedra@utad.pt; 3 CITAB—Centro de Investigação e Tecnologias Agroambientais e Biológicas, Universidade de Trás-os-Montes e Alto Douro, Quinta de Prados, 5000-801 Vila Real, Portugal; 4 CECAV—Centro de Ciência Animal e Veterinária, Universidade de Trás-os-Montes e Alto Douro, P.O. Box 1013, 5001-801 Vila Real, Portugal; 5 Epicore Networks Inc., 4 Lina Lane, Eastampton, Burlington, NJ 08060, USA; Russell.Jerusik@adm.com; 6 Departamento de Ciências Veterinárias, ECAV, Universidade de Trás-os-Montes e Alto Douro, Quinta de Prados, 5000-801 Vila Real, Portugal

**Keywords:** fish diseases, quorum-quenching, *Bacillus* spp., zebrafish larvae

## Abstract

The disruption of pathogen communication or quorum-sensing (QS) via quorum-quenching (QQ) molecules has been proposed as a promising strategy to fight bacterial infections. *Bacillus* spp. have recognizable biotechnology applications, namely as probiotic health-promoting agents or as a source of natural antimicrobial molecules, including QQ molecules. This study characterized the QQ potential of 200 *Bacillus* spp., isolated from the gut of different aquaculture fish species, to suppress fish pathogens QS. Approximately 12% of the tested *Bacillus* spp. fish isolates (FI). were able to interfere with synthetic QS molecules. Ten isolates were further selected as producers of extracellular QQ-molecules and their QQ capacity was evaluated against the QS of important aquaculture bacterial pathogens, namely *Aeromonas* spp., *Vibrio* spp., *Photobacterium damselae*, *Edwardsiela tarda*, and *Shigella sonnei*. The results revealed that *A. veronii* and *E. tarda* produce QS molecules that are detectable by the *Chr. violaceum* biosensor, and which were degraded when exposed to the extracellular extracts of three FI isolates. Moreover, the same isolates, identified as *B. subtilis*, *B. vezelensis*, and *B. pumilus*, significantly reduced the pathogenicity of *E. tarda* in zebrafish larvae, increasing its survival by 50%. Taken together, these results identified three *Bacillus* spp. capable of extracellularly quenching aquaculture pathogen communication, and thus become a promising source of bioactive molecules for use in the biocontrol of aquaculture bacterial diseases.

## 1. Introduction

Despite experiencing continuous growth, the development of aquaculture remains highly vulnerable to the occurrence of infectious bacterial diseases. Furunculosis, edwardsielosis, vibriosis, and photobacteriosis are among the most prevalent diseases, with serious effects on marine fish species [1]. Although antibiotics are an important tool for disease treatment, their damaging effects on the environment and public health have led to increased restrictions on their use in aquaculture [2]. An alternative to antibiotics relies on attenuating pathogen virulence, a process called anti-virulence therapy [3].

Virulence factors (e.g., motility, extracellular polysaccharides, biofilms, lytic enzymes, or secretion systems) enable bacterial pathogens to colonize and damage their host. Their expression can be regulated through complex regulatory networks, including Quorum-Sensing (QS) [4]. QS is a process of cell-to-cell communication in which bacteria coordinate their population size and behavior [5,6]. It is mediated by small diffusible signals, known as autoinducers, that are produced and exported from the cell, and then perceived by specific receptors in the bacterial community, improving their global fitness [6]. QS can occur both within and between bacterial species. The first QS system model relied on the identification of acyl-homoserine lactones (AHLs) as regulators of bioluminescence production in *Vibrio fischeri* [7]. In brief, AHLs are synthetized by LuxI homolog proteins (AhyI, AsaI, EdwI in *A. hydrophila*, *A. salmonicida* and *E. tarda*, respectively) that diffuse out/into the cell, where the LuxR homolog (AhyR, AsaR, EdwR in *A. hydrophila*, *A. salmonicida* and *E. tarda*, respectively) detects these molecules and binds to the promoter of QS-dependent target genes [6]. 

Although AHLs are the most common signal molecules in Gram-negative bacteria (including fish pathogens), bacteria can produce and detect other signal molecules involved in gene-virulence regulation, such as autoinducer-2 (AI-2) and cholera autoinducer-1 (CAI-1) [8]. In *Vibrio anguillarum*, *V. harveyi*, and *V. parahaemolyticus*, these three types of signal molecules were identified to regulate the expression of genes encoding proteases, toxins, and other virulence-associated factors [9,10]. *Aeromonas hydrophila*, *V. vulnificus*, and *Edwardsiella tarda* use a combination of several AHLs with AI-2 to regulate motility, biofilm formation, proteases, hemolysin, and secretion systems [8,11], whereas *A. salmonicida* and *Tenacibaculum maritimum* were reported to only use AHLs as signal molecules [12,13].

Considering the key role of QS in bacterial communities’ fitness, one of the most-studied anti-virulence therapy strategies is the disruption of QS, called Quorum-Quenching (QQ). Compared to bactericidal/bacteriostatic approaches, QQ is unlikely to promote strong selective pressures, minimizing the development of resistance among pathogens, since it does not directly target bacterial growth [4,14]. QQ can be achieved by (i) inhibiting the signal biosynthesis, (ii) enzymatically degrading/modifying the signal molecules, or (iii) inhibiting signal detection by blocking the signal receptor [15]. Enzymatic degradation of the signal is the most-studied approach in QQ and appears to be widely distributed in the bacterial kingdom. Thus, the majority of QQ enzymes were discovered in bacteria, targeting AHLs signal molecules [14], namely, AHL lactonases, acylases, or oxi-reductases, depending on the molecule modification. By degrading the QS signal, these enzymes can decrease the production of virulence factors in pathogenic bacteria. The first identified QQ enzyme was an AHL-lactonase (encoded by *aiiA*) from *Bacillus* sp. 240B1 that, when tested in vivo, increased plant disease resistance by attenuating *Erwinia carotovora* virulence [16]. Other QQ enzyme-encoding genes were discovered, such as *attM* (*Agrobacterium tumefaciens*) [17], *ahlD* (*Arthrobacter* sp.) [18], *aiiM* (*Microbacterium* sp.) [19], and *ytnP* (*B. subtilis*) [20]. 

*Bacillus* spp. are spore-forming, gram-positive bacteria with recognizable biotechnology applications, including as probiotic-health-promoting agents or as a source of antimicrobial molecules with the capacity to inhibit fish pathogens [21,22,23,24,25,26,27]. Indeed, from the gut of marine fish species, we recently isolated several *Bacillus* spp. strains capable of inhibiting the growth and biofilm formation of 14 different fish pathogens with in vitro QQ capacity [27]. Recent reports suggest that *Bacillus* spp. with QQ activity can reduce pathogens’ QS and virulence, increasing fish protection against *A. hydrophila* and *Vibrio* spp.: (i) *B. licheniformis* T1 and *Bacillus* sp. AI96 QQ increased zebrafish resistance to *A. hydrophila* by 50% and 40%, respectively [28,29]; (ii) *Bacillus* sp. QSI-1 increased zebrafish survival by 70% when challenged with *A. hydrophila* [30]; (iii) The QQ strain *Bacillus* sp. YB1701 improved *Carassius auratus gibelio* resistance to *A. hydrophila* by 25% [31]; (iv) *B. licheniformis* DAHB1 AiiA enzyme and *Bacillus* sp. NFMI-C protected shrimp and prawn larvae against *V. parahaemolyticus* and *V. campbellii* infection, respectively [32,33]; (v) *B. thuringiensis* and *B. cereus* strains showed QQ and protective activities against *Y. ruckeri* in rainbow trout [34]. 

This study screened the QQ capacity of the extracellular components of a collection of *Bacillus* spp., which were previously isolated from the gut of different marine fish species, and tested their ability to degrade the AHLs signals produced by important fish pathogenic strains. After validation of fish isolates (FIs) with QQ capacity, their potential protective effects were determined in an in vivo model (zebrafish larvae) when challenged with *E. tarda*. 

## 2. Results

### 2.1. Fish-Isolates Produce Extracellular Compounds with QQ Capacity

**Figure 1:**
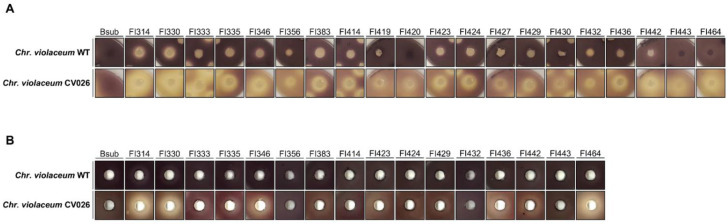
Violacein pigment production by *Chr. violaceum* biosensors when exposed to sporeforming fish isolates. (**A**) Inhibition of biosensor’s violacein pigment production by FI isolates (FI numbers on top). (**B**) Biosensor’s violacein pigment inhibition by the cell-free supernatant of sporeforming fish isolates (FI numbers on top). All photos were taken with a Sony IMX240 camera and are at the same scale.

Around 200 fish-isolates (FI) were tested for their quorum-quenching activity (QQ) of the broad range AHLs (N-(3hydroxydecanoyl)-L-homoserine lactone (3-hydroxy-C10HSL), N-octanoyl-L-Homoserine lactone (C8-HSL), and other minor AHLs) produced by the wild-type biosensor *Chromobacterium violaceum* (CECT 494, ATCC 12472) [35] or of the N-(3-Oxohexanoyl)-L-homoserine lactone (3-Oxo-C6-HSL), supplemented with the CV026 biosensor, a *Chr. violaceum CviI*-negative mutant in which violacein production can be restored in the presence of exogenous AHLs [36]. The inactivation of AHL compounds was confirmed by the loss of purple violacein pigmentation in the QQ biosensors around FI colonies (Figure 1A). The screenings revealed that out of the 200 FI tested, 11 (5.5%) displayed activity against AHLs produced by the wild-type biosensor, and 29 (14.5%) degraded the 3-oxo-C6-HSL signalling molecule. When using a CV026 biosensor supplemented with 3-Oxo-C6-HSL, 18 FI produced a clear pigment inhibition halo (Figure 1A), indicating that all isolates could inhibit 3-Oxo-C6-HSL-like molecules, but when using the wild-type biosensor, only isolates FI314, FI330, FI335, FI346, FI383, FI414, FI423, FI424, FI432, FI436, and FI442 could interfere with the several AHLs that were produced (Figure 1A). 

When testing their cell-free supernatants (to establish the intra- or extracellular localization of the QQ compounds), it became clear that FI did not produce extracellular compounds capable of interfering with all AHLs of the wild-type biosensor (Figure 1B). In contrast, the inhibition of violacein pigment production was observed in the CV026 biosensor supplemented with 5 µM of 3-Oxo-C6-HSL, and was particularly evident in FI314, FI330, and FI464, which showed stronger activity when compared to the other isolates (Figure 1B). These results indicate that FI isolates produce extracellular compounds with QQ capacity against at least 3-Oxo-C6-HSL QS molecules. The laboratory strain *B. subtilis* 168 could not inhibit pigment production of the biosensors in both bioassays.

### 2.2. Isolates QQ Activity Is Mediated through AHLs Enzymatic Inactivation

**Figure 2:**
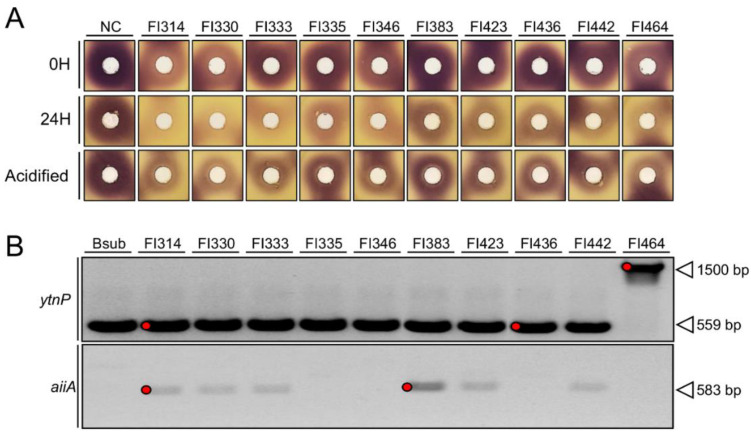
Activity and genomic detection of putative QQ lactonases. (**A**) Enzymatic degradation of 3-Oxo-C6-HSL by the fish isolates’ extracellular compounds (FIs on the top), followed by reversion of the enzymatic reaction through acidification, revealed by the reduction and restoration of the violacein pigment production by the biosensor. All photos were taken with a Sony IMX240 camera and are at the same scale. (**B**) PCR detection of genes coding for a putative QQ lactonase (*ytnP*) and N-acyl homoserine lactonase (*aiiA*) in the genomes of *B. subtilis* 168 (Bsub) and fish isolates (FI numbers on top). The amplicon size, in base pairs (bp), is shown on the right. The figure was constructed using different zones of the agarose gel. PCR products marked with a red circle in the figure were sequenced using the corresponding forward and reverse primers (Table 1).

To evaluate the catalyst nature of the QQ activity observed in the previous experiment, the extracellular compounds of all positive strains (FI314, FI330, FI333, FI335, FI346, FI383, FI423, FI436, FI442, and FI464) were mixed for 24 h with 30 µM of synthetic 3-Oxo-C6-HSL, before a bioassay with CV026 biosensor. In this, the reduction in the violacein halo when submitted to the H24 reaction mixture, when compared to a fresh reaction mixture (H0), indicates lactone degradation by the extracellular compounds present on the FI supernatants, and thus an enzymatic reaction. The FI extracellular compounds mixed with the 3-Oxo-C6-HSL led to a reduction in pigment production by the biosensor in all combinations, with strains FI314, FI330, FI346, and FI464 showing the highest activity (Figure 2A). A reduction in the pigment production was also noticed when using the fresh (0H) reactions of strains FI314, FI330, and FI464, which might indicate the presence of signal blockers (Figure 2A) on their supernatant. 

To clarify if the degradation of the 3-Oxo-C6-HSL could be due to lactonase, FI extracellular compounds were simultaneously tested for lactonase enzymatic activity through acidification, since lactonase enzymatic reaction is pH-mediated and reversible by acidification. After the acidification process, the enzymatic reaction could be reversed, as confirmed by the regain of purple pigmentation intensity, suggesting a lactonase-type FI QQ activity (Figure 2A). As expected, the negative control (LB medium mixed with 30 µM of 3-Oxo-C6-HSL) did not reduce pigment production during the bioassay (Figure 2A).

To elucidate the lactonases behind the observed QQ activities, all 10 FI isolates were first identified based on partial sequencing of the 16S rRNA gene (~1000 bp), as part of the *Bacillus* genera. FI314, FI330, FI346, and FI442 were identified as *B. subtilis*; FI333, and FI423 as *B. amyloliquefaciens;* FI335, FI383, and FI436 as *B. velezensis*; and FI464 as a *B. pumilus* (Supplementary Table S1). The presence of genes that were previously correlated with QQ activity in *Bacillus* species was first investigated using oligonucleotide primers described by Kalia, et al. [37] to amplify the Firmicutes AHL-lactonase gene *aiiA* (Table 1). Since no amplification could be obtained in the target FI (data not shown), novel oligonucleotide primers were specifically designed in highly conserved genomic regions to target the genes of interest: *aiiA* (N-acyl homoserine lactonase) and *ytnP* (probable quorum-quenching lactonase). As shown in Figure 2B, all strains except FI464 showed a PCR band with the expected size (559 bp) of *ytnP* gene. In isolate FI464, a strong PCR band was observed at a higher molecular weight (~1500 bp). However, only faint bands with the expected size (583 bp) of *aiiA* could be detected in FI314, FI330, FI333, FI383, FI423, and FI442. 

Conjugating the previously observed genomic profile and QQ bioactivities, five PCR products were sequenced to confirm if they matched the targeted gene (bands marked in red in Figure 2B). All DNA sequences could be assigned to a protein family using BLASTx (https://blast.ncbi.nlm.nih.gov/Blast.cgi; accessed on 15 January 2020) (Table 2). The amino acid sequences of putative AHL-lactonases were aligned with other known AHL-lactonases, and the conserved motif “HXHXDH” present in the metallo-β-lactamase superfamily was searched for, as illustrated in Supplementary Figure S1A. Putative *ytnP*-like AHL-lactonases from FI314 and FI436 have the zinc-binding motif “HXHXDH” (Supplementary Figure S1A) and cluster with the *B. subtilis* 168 *ytnP* (Supplementary Figure S1B), being identified through BlastX as *ytnP*-like metallo-hydrolase and MBL fold metallo-hydrolase, respectively. However, the high-molecular-weight band of FI464 did not correspond to a putative QQ lactonase but to a DAK2 domain-containing protein (Table 2) and did not cluster to any QQ enzymes known in *Bacillus* spp. (Supplementary Figure S1B). Putative AHL-lactonase *aiiA*, from strains FI314 and FI383, clustered separately from the other AHL-lactonases in the metallo-β-lactamase family, and were identified as containing a rhodanese-like homology domain (Table 2).

### 2.3. Isolates QQ Compounds Can Interfere with Fish Pathogens AHLs

After establishing the putative lactonase type of FI QQ activity, FI QQ compounds were tested for interference with fish pathogens AHLs. For this, 13 different fish pathogens (Table 3) were studied for the production of AHLs’ detectable by *Chr. violaceum* CV026 biosensor. From the 13 tested pathogens, only *Aeromonas salmonicida*, *A. veronii*, *A. bivalvium*, and *Edwardsiella tarda* could induce the production of a violacein pigment on the biosensor by cross-feeding (Supplementary Figure S2). 

**Figure 3:**
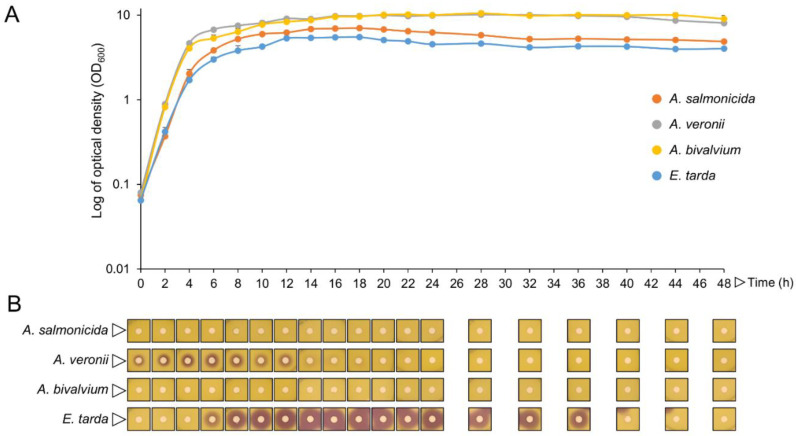
Growth curves and AHLs production kinetics of *A. salmonicida*, *A. veronii*, *A. bivalvium* and *E. tarda*. (**A**) Optical density of bacterial pathogens grown for 48 h at 25 °C (or 37 °C in the case of *E. tarda*), 140 rpm. (**B**) Violacein pigmentation halos of *Chr. violaceum* CV026 around the wells filled with filtered cell-free supernatant from the pathogens’ bacterial cultures at different timepoints. The data are composed of three independent experiments. All photos were taken with a Sony IMX240 camera and are at the same scale.

As AHLs can diffuse through the cell, their production kinetics and cell-release were investigated on those four pathogens. By using pathogens’ extracellular medium in a well-diffusion method, it was observed that the production of AHLs by *A. veronii* started at the beginning of its growth curve, lasting for 12 h, with a maximum peak at the transition from the late exponential to the early stationary phase of growth (Figure 3A,B). In *E. tarda*, AHLs detection started at the transition from the exponential to stationary growth phase and was extended until 36 h of growth (Figure 3A,B). Although observable by cross-feeding (Supplementary Figure S2), the use of an extracellular medium in the well-diffusion method did not allow for the detection of AHLs produced by *A. salmonicida* and *A. bivalvium* by the CV026 biosensor at 48 h of the assay (Figure 3B). 

Before ascertaining the FI QQ capacity against natural AHLs from fish pathogens, the QQ kinetics of FI314, FI436, and FI464 (selected by conjugating the previous QQ bioassays, molecular identification, and the QQ genomic profile) was assessed using synthetic 3-oxo-C6-HSL. For all strains, the maximum production of QQ compounds was during the early stationary growth phase (~8 h) (Supplementary Figure S3).

**Figure 4:**
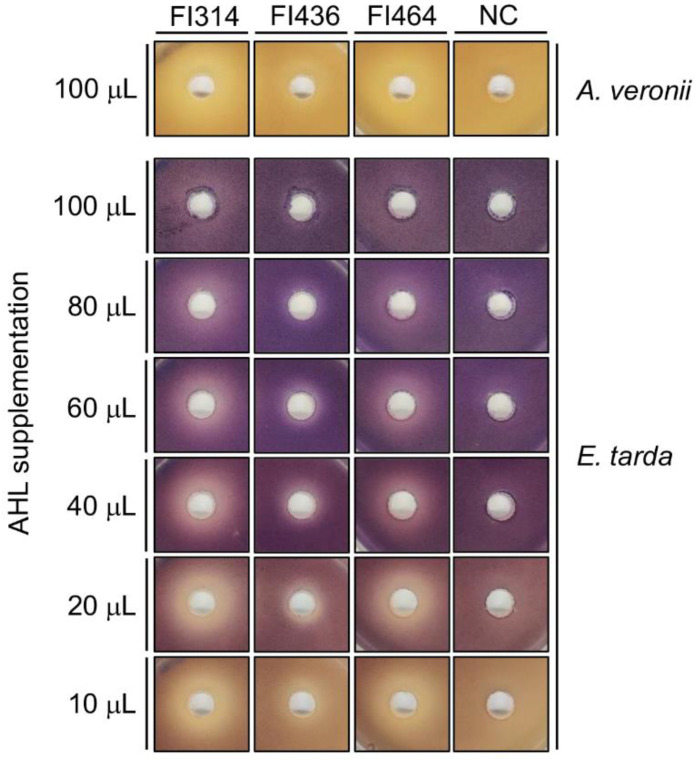
Inhibition of *A. veronii* and *E. tarda* AHLs’ detection by the *Chr. violaceum* CV026 biosensor. Biosensor’s violacein pigment production when supplemented with natural AHLs extracted from *A. veronii* and *E. tarda* (10, 20, 40, 60, 80 and 100 μL) around wells containing cell-free supernatant of sporeforming fish isolates (FI numbers on top). All photos were taken with a Sony IMX240 camera and are at the same scale.

Fish pathogens AHLs were extracted from the cell-free supernatants of 6 h and 14 h cultures of *A. veronii* and *E. tarda*, respectively, and analyzed through agar well-diffusion assay using different supplementation amounts (10–100 µL in 8 mL of soft-agar) (Figure 4). *A. veronii* crude AHL extracts induced a slight violacein pigmentation on CV026 biosensor with 100 μL of supplementation. The AHL crude extract from *E. tarda* induced purple pigmentation on the biosensor CV026 with 10–100 μL of supplementation. As illustrated in Figure 4, all three selected FI strains could inactivate the signals produced by *A. veronii* and *E. tarda*, with FI314 and FI464 showing a higher QQ potential. Additionally, a decrease in QQ activity could also be observed with an increase in *E. tarda* AHLs supplementation (Figure 4), indicating a concentration-dependent activity. 

### 2.4. Isolates QQ Compounds Protect Zebrafish Larvae upon E. tarda Challenge

The next question was whether the in vitro FI QQ activity against *E. tarda* AHLs could originate from an in vivo protection of FI strains to zebrafish larvae challenged with *E. tarda*. 

For this, the maximum non-toxic extract concentration (MNTC) of each FI to be employed in protective assays was first established using 4 dpf zebrafish larvae (Supplementary Figure S4A). The results showed that extracts from all strains induced toxicity in zebrafish larvae when administrated at concentrations above 250 μg mL^−1^ and, thus, FI extracts were used at 250 μg mL^−1^ (MNTC). This concentration did not influence *E. tarda* growth (data not shown). 

Secondly, an *E. tarda* infection model was established by exposing 10 dpf zebrafish larvae by immersion with 5 × 10^7^, 1 × 10^8,^ and 3 × 10^8^ CFU mL^−1^ of *E. tarda* for 24 h. Larvae exposed to 3 × 10^8^ CFU *E.tarda* mL^−1^ started to show mortality at 10 h post-infection (hpi) and rapidly progressed until 18 hpi, with 100% mortality (data not shown and Supplementary Figure S4). Mortalities in larvae exposed to 1 × 10^8^ CFU *E. tarda* mL^−1^ began at 17 hpi and progressed through time, reaching ~60% at 24 hpi. On the other hand, 5 × 10^7^ CFU *E. tarda* mL^−1^ started to induce mortalities only at 23 hpi (Supplementary Figure S4B). Control larvae did not exhibit any mortality throughout the experimental trial. From the overall results, 1 × 10^8^ CFU *E. tarda* mL^−1^ was selected as the bacterial concentration for the challenge experiment.

**Figure 5:**
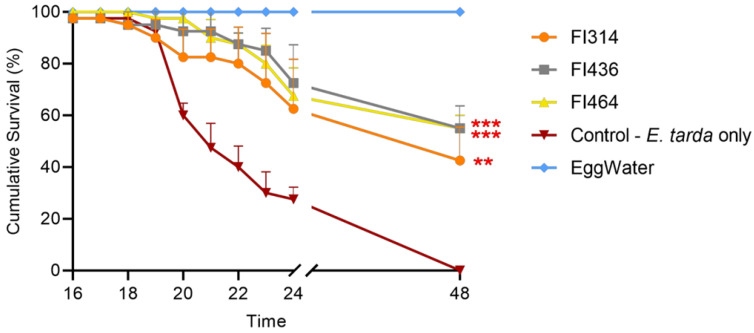
FI extracts protection of zebrafish larvae against infection with *E. tarda*. 7 dpf zebrafish larvae were immersed for 2 h with 250 μg mL^−1^ of each FI extract and three days later, challenged with *E. tarda* at a final concentration of 1 × 10^8^ CFUs mL^−1^ for 24 h. Untreated larvae challenged with *E. tarda* and, untreated and unchallenged larvae were used as positive and negative control, respectively. Data are composed of three independent experiments. Significant differences (*p* < 0.01; *p* < 0.001) in relation to control are represented by asterisks (**, ***, respectively).

Finally, the protective effect of FI extracellular compounds was evaluated using the *E. tarda* infection model. As illustrated in Figure 5, FI extracellular compounds from FI314, FI436, and FI464 were able to protect zebrafish larvae from *E. tarda* infection, significantly increasing larvae survival rate when compared to the control (non-treated zebrafish larvae infected with *E. tarda*). In the control group, at 24 hpi, only 30% of zebrafish larvae survived, which resulted in 100% mortality at 48 hpi. By comparing treatments with the control group, after 48 hpi, FI314 increased the average survival rate of challenged larvae by 43% (*p* < 0.01), and strains FI436 and FI464 increased the survival rate upon challenge by 50% (*p* < 0.001). 

## 3. Discussion

*Bacillus* spp. have been extensively studied for their use in aquaculture due to their probiotic attributes, which include the production of bioactive compounds and the modulation of the host immune response [40]. In addition to their well-known antimicrobial activity, *Bacillus* spp. are also producers of quorum-quenching (QQ) molecules [27,29,41]. The disruption of pathogens’ communication or quorum-sensing via QQ molecules has been proposed as a promising alternative to fighting bacterial infections in aquaculture [15], which remains a major constraint to the sustainable development of the sector. We recently described the potential of fish-gut *Bacillus* spp. isolates (FI) as a source of QQ molecules [27]. In this study, we took advantage of 200 FI isolated from the gut of different fish species (*Sparus aurata*, *Dicentrarchus labrax*, and *Diplodus sargus*) [24,27] to further explore their QQ potential, by testing their ability to partially or completely degrade AHLs’ QS molecules used by gram-negative fish pathogens. Consistent with the literature, the results indicate that fish gut *Bacillus* spp. have QQ potential and, by testing their extracellular compounds, the majority of these *Bacillus* spp. produce and release QQ compounds to the extracellular environment. This observation contradicts previous reports that described AHL-degrading activities in *Bacillus* spp. as cytoplasmatic or cell-wall-associated [14,16,42] and might represent a technological advantage for QQ disease control, since compounds that work extracellularly are believed to exert less selective pressure in evolving resistance among pathogens [3,14,43]. 

AHLs can be degraded through the enzymatic activity of lactonases, acylases, or oxi-reductases. To date, mainly lactonases, but also one oxidoreductase and one putative acylase, have been described in *Bacillus* species [42,44,45,46]. When an AHL-lactonase is present, cleavage of the homoserine lactone ring of the AHL molecule occurs. The opening of the lactone ring makes the AHL molecule incapable of binding to the target transcriptional regulators (e.g VanT, AhyR, and LuxR homologs), attenuating its effectiveness and detection by the biosensor [47]. This hydrolysis is pH-mediated and can be reversed by acidification. Taking this into consideration, the extracellular enzymatic activity of the positive FI isolates was tested, and all 10 strains enzymatically degraded the AHLs after 24 h of incubation, with a partial restoration of the AHL molecule, inferred by the recovery of the violacein pigment on the biosensor during the acidification process. The restoration of the violacein pigment on the biosensor after the acidification process indicated the putative presence of an AHL-lactonase. However, pigment regain was only partial, which may be explained by the lack of pH control during incubation, which is essential to the hydrolysis reversion of the lactone ring [48]. Although the presence of an acylase, an oxidoreductase, or small molecules that blocked the biosensor signal receptors cannot be ruled out, AHLs degradation seen by the smaller colour formation on the biosensor submitted to the 24 h AHL-SUP mixture when compared to the colour observed on the biosensor submitted to fresh (H0) AHL-SUP mixture, followed by colour restoration upon acidification, indicate that the observable QQ activity is probably due to a lactonase-like enzyme. 

In the lactonase-like enzyme family, the *aiiA* gene and AiiA enzyme were the first to be discovered and characterized for their QQ activity in *Bacillus* spp. [16]. Over the years, different QQ studies highlighted the potential of the AiiA enzyme in the prevention of plant and animal bacterial infections [16,28,49]. In the present study, attempts to amplify the *aiiA* gene, both using the literature-described primers and primers designed by us, were unsuccessful. Despite the presence of light PCR bands in the agarose gel (using the primers designed in this study), the analysis of their nucleotide and translated sequences did not correspond to the *aiiA* gene or contain the AHL-lactonases’ conserved zinc-binding motif "HxHxDH" [42]. Although Metallo-β-lactamase family members possess the same folding and conserved sequences, the group englobes proteins with divergent sequences and biological functions [50]. Thus, it was hypothesized that, by using the primers designed in homologous sequences, other Metallo-β-lactamase proteins were amplified, with no similarity to the *aiiA* gene. In fact, by considering the taxonomic identification of the QQ strains, only a few studies have reported the presence of the *aiiA* gene in *B. subtilis* and *B. amyloliquefaciens* [49,51], and none in *B. vezelensis* and *B. pumilus*.

Meanwhile, Schneider, Yepes, Garcia-Betancur, Westedt, Mielich and López [20] reported the expression of another QQ gene, the *ytnP* from *B. subtilis*, capable of interfering with the signaling pathways of biofilm formation and streptomycin production in *Pseudomonas aeruginosa* and *Streptomyces griseus*. Here, the *ytnP* gene was successfully amplified in 9 out of the 10 QQ strains. Protein sequence analysis of FI314 and FI436 showed similarity with *ytnP*-like and MBL fold proteins, respectively, and revealed the presence of the AHL-lactonase zinc-binding motif containing the key histidine residues “HxHxDH” of the lactonase architecture [42]. The YtnP enzyme has been described to accumulate in the cytoplasm, and only upon the presence of other antimicrobials and stress factors [20]. In the strains used in this study, the observed QQ activity was extracellular, and is thus unlikely to only be due to the action of YtnP. An additional undescribed QQ lactonase, or at least a differently regulated *ytnP*, must be present. The deletion of *ytnP* in the FI isolates background could provide a glimpse into their QQ mechanism, but transformation of these strains (both by chemical methods and by electroporation) has been unsuccessful to date (data not shown). 

Additionally, despite showing a putative AHL-lactonase activity in the biochemical test, none of the test QQ genes nor the conserved motif of AHL-lactonase were amplified from FI464, identified as *B. pumilus*. In fact, to the authors’ knowledge, there is only one report describing *B. pumilus* QQ potential. Nithya, Aravindraja and Pandian [46] reported a *B. pumilus* strain with a putative acylase activity, which effectively reduced biofilm formation and other virulence factors in *P. aeruginosa*. However, the authors did not characterize the protein in detail or provide the gene sequence. Thus, the FI464 genome is undergoing further investigation, as it is assumed that this strain possesses a completely different lactonase activity to the one described in the literature for the other *Bacillus* species. 

The ability of the FI to interfere with fish pathogens QS was the core of this study. Thus, the three strains with the best QQ profile, FI314, FI436, and FI464, were evaluated for their ability to degrade the natural signals produced by different species of problematic fish pathogens. QS systems based on AHLs are well-described and reviewed in fish bacterial pathogens [8,12,13,52,53,54,55,56,57,58]. Here, AHLs’ production was evaluated by cross-feeding fourteen different fish pathogens, including species from *Aeromonas*, *Vibrio*, *Photobacterium*, *Edwardsiella*, and *Shigella* genera, with the biosensor *Chr. violaceum* CV026. Of the tested pathogens, only *A. salmonicida*, *A. veronii*, *A. bivalvium*, and *E. tarda* had the capacity to induce violacein pigmentation on the biosensor *Chr. violaceum* CV026. This biosensor has the limitation of being stimulated by AHLs with acyl chains ranging from C4 to C8 and, when these are present alongside long-acyl-chain molecules (C8-C14), inhibition of violacein production occurs [36]. This might explain the lack of violacein induction on the biosensor by the tested *Vibrio* spp., which are known to produce several QS molecules, including AHLs with different lengths and derivates, such as C4-HSL, C6-HSL, 3-oxo-C10-HSL, 3-oxo-C14-HSL [9,56,57,59]. Bruhn, Dalsgaard, Nielsen, Buchholtz, Larsen and Gram [52] also reported a lack of violacein induction on the CV026 biosensor when testing *V. anguillarum*, *V. vulnificus*, and *Photobacterium damselae* subsp. *damselae* strains, but demonstrated AHLs production in *Vibrio* spp. using another biosensor. 

*E. tarda* and *Aeromonas* spp. have previously been reported to induce the violacein pigment on the CV026 biosensor [12,54,55,60]. The exception in these results, as anticipated, was the *A. hydrophila* LMG 2844 strain, as it was unable to induce an AHL-mediating response that was detectable by different biosensors, including CV026 [60,61]. An unexpected result was the lack of violacein pigment when testing *T. maritimum*, since Romero, Avendano-Herrera, Magarinos, Camara and Otero [13] reported that this species produces C4-HSL. Although the CV026 biosensor detect Ced4-HSL molecules, no activity was reported in the Flavobacteriaceae family using this biosensor [52], and, to the authors’ knowledge, there is no study reporting violacein induction on a CV026 biosensor by *T. maritimum* AHLs. Moreover, genome sequencing of the *T. maritimum* strain NCIMB 2154^T^ revealed the absence of homologous genes for AHLs synthesis [62].

Next, the pathogens that induced violacein pigmentation on the *Chr. Violaceum* biosensor, i.e., *A. salmonicida*, *A. veronii*, *A. bivalvium*, and *E. tarda*, were tested for the maximum peak in AHLs production and extracellular accumulation. Although *A. salmonicida* and *A. bivalvium* induced the violacein pigment on the cross-feeding bioassays, they failed to induce it when testing the extracellular accumulation for 48 h. This might be due to low AHLs concentration or stability outside the cell. *A. veronii* accumulated AHLs during the whole exponential phase with a maximum peak at the time of transition to the stationary phase, followed by a rapid decline, as described by Jangid, et al. [63]. However, AHLs accumulation in *E. tarda* was only detected when the cells were entering the stationary phase, and was stable throughout this phase, with the maximum peak detected at 16 h of growth. Accordingly, Han, Li, Qi, Zhang and Bossier [55] also detected AHLs production during the *E. tarda* growth phase (1–21 h) using the *Ag. tumefaciens* KYC55 biosensor and, since this biosensor is considered to be ultra-sensitive in AHLs detection, the accumulation of AHLs was detected 5 h earlier. 

*A. veronii* is known to produce natural short and medium acyl-chains, such as C6-HSL, C8-HSL, 3-oxo-C8-HSL, and 3-hydro-C8-HSL [64]. In CV026, C8 acyl chains led to an inhibition of violacein induction by other molecules, such as C6-HSL [36]. This might explain the minor induction of violacein pigment on the biosensor, observed when the crude and concentrated *A. veronii* extracts were used. Nonetheless, FI314 and FI464 were able to interfere with these natural AHLs, either through enzymatical degradation or by interrupting their detection by bacterial receptors. When testing different quantities of *E. tarda* AHLs supplementation, it could be observed that an increase in supplementation led to a decrease in QQ activity, which might indicate an enzymatic degradation of C4-HSL, C6-HSL, 3-oxo-C6-HSL, and C7-HSL [54,55] by all the tested FIs. Recent studies have highlighted the QQ potential of *Bacillus* spp. against natural AHLs produced by fish pathogens such as *A. hydrophila* [30,65,66], *Yersinia ruckeri* [34], and *V. harveyi* and *V. alginolyticus* [41]. Similarly, Gui, Wu, Liu, Wang, Zhang and Li [53] demonstrated that a purified lactonase *aiiA_AI96_* from *Bacillus* sp. enzymatically inactivated the AHLs produced by *A. veronii*, as well as other QS-controlled behaviors. To the authors’ knowledge, to date, only Romero, et al. [67] have described an in vitro quenching of *E. tarda* AHLs (C6-HSL and 3-oxo-C6-HSL) using *Tenacibaculum* sp. strain 20J cell extracts. Thus, this is the first report demonstrating the potential of QQ *Bacillus* spp. in inhibiting the natural AHLs produced by *E. tarda.*

*E. tarda* is an important bacterial pathogen that causes hemorrhagic septicemia, edwardsielosis, affecting economically important aquaculture fish species such as turbot [68], Senegalese sole [69], and tilapia [70,71,72], and has been associated with gastro- and extraintestinal infections in humans [73,74]. 

*Bacillus* QQ strains have been used in several in vivo studies for fish disease mitigation, but, to date, none has addressed *E. tarda* infections. For example, oral administration of purified QQ enzyme AiiA increased zebrafish survival by 40% when challenged with *A. hydrophila* [28], and increased shrimp survival by 50% when challenged with *V. parahaemolyticus* [33]. Similarly, when cells of QQ *B. cereus* and *B. thuringiensis* strains were fed to rainbow trout, fish survival upon infection with *Y. ruckeri* increased by 80% [34]. Additionally, Chen, et al. [75] co-injected a purified QQ enzyme (AiiA B546) with *A. hydrophila*, reducing common carp mortality by 25%, and, in a similar experiment, cells of the QQ *B. licheniformis* T-1 strains decreased zebrafish mortality by 50% when co-injected with *A. hydrophila* [29]. 

In the present study, the treatment of zebrafish larvae with FI314, FI436, and FI464 extracts significantly reduced their mortality when challenged with *E. tarda* by immersion. In *E. tarda*, the routes of infection are believed to be the gut, the skin, and the gills [76]. The gills and the skin are in constant contact with the environment and, consequently, are accessible to pathogen entry. Thus, the presence of QQ molecules at these sites may help prevent or delay fish infection. On the other hand, in the present study, there was no physical contact between the FIs extracts and *E. tarda*, suggesting that these molecules can circulate through the host, exerting their protective effect. Although FIs extracts did not inhibit *E. tarda* growth (data not shown), a possible direct stimulus in the larvae immune system, facilitating disease resistance, cannot be ruled out. This interpretation is only speculative and requires further investigation, including the determination of a putative stimulus in the fish immune system using such extracts. Importantly, the extracts showed good stability (up to 12 months) under no specific storage conditions (room temperature). Moreover, a small bacterial culture volume (200 mL) allowed lyophilized extract to be obtained, make up to 40 L of treating water. These characteristics reinforce the practical application of these extracts in the aquaculture industry. 

As a conclusion, this work describes three *Bacillus* spp. which are capable of extracellular quenching *E. tarda* AHLs while protecting the model zebrafish larvae from infection. The lack of studies regarding *E. tarda* infection and its crescent impact in aquaculture emphasizes the novelty and importance of this study. Nonetheless, further studies are required to fully characterize the QQ molecules responsible for the bioactivities described and to clarify the QQ mechanism involved. QS plays an important role in bacterial pathogenesis and virulence; thus, the QQ molecules from FI314 (*B. subtilis*), FI436 (*B. vezelensis*), and FI464 (*B. pumilus*) may be promising tools for disease control in aquaculture.

## 4. Materials and Methods

### 4.1. Bacterial Strains and Culture Conditions

Bacterial strains used in this study are listed in Table 3. Fish-Isolates (FI) from our collection obtained from the gut of different fish species (*Sparus aurata*, *Dicentrarchus labrax*, and *Diplodus sargus*) [24,27], and the laboratory strain *Bacillus subtilis* 168 [77] used as control for FI growth, were routinely and aerobically grown in Luria–Bertani (LB; Fisher BioReagents, Waltham, MA, U.S.A) medium at 37 °C. QQ biosensor strains, *Chromobacterium violaceum* (CECT 494, ATCC 12472) and *Chr. violaceum* CV026 (CECT 5999) (Table 1), were grown aerobically in LB medium at 30 °C. *Chr. violaceum* CV026 is a *cvil*::mini-Tn5 mutant requiring kanamycin (25 μg mL^−1^) (Nzytech, Lisboa, Portugal) supplementation. Fish pathogens were grown aerobically in Brain Heart Infusion (BHI, BD Difco) medium (except for *T. maritimum*, which was grown in marine medium (BD Difco)) at 25 °C or 37 °C (for *E. tarda*, *S. sonnei*). 

### 4.2. Evaluation of Isolates QQ Activity

A flow diagram with the methodology used for evaluating QQ activity is presented in Supplementary Figure S5. 

QQ potential of 200 FI was first evaluated with overnight cultures of each FI inoculated as 5 μL spot on LB agar plates and incubated for 24 h at 37 °C, before killing with chloroform vapours. Plates were aerated, before overlaying with LB soft agar (0.8% agar) inoculated with *Chr. violaceum* WT (OD_600_~0.1), or with *Chr. violaceum* CV026 (OD_600_~0.1) supplemented with 5 µM of N-(β-Ketocaproyl)-L-Homoserine Lactone (3-Oxo-C6-HSL) (Sigma-Aldrich, Darmstadt, Germany). Zones of violacein pigmentation inhibition around the *Bacillus* sp. colonies (without interference on the biosensor growth) after 48 h at 30 °C were considered a positive result for QQ activity. 

The extracellular QQ activity was measured in all positive strains from the previous assay. For that, cell-free supernatant of each FI was prepared from overnight cultures grown at 37 °C and 140 rpm, followed by centrifugation for 10 min at 13,000× *g* and sterilization by filtration with 0.22 µm cellulose acetate filter. LB agar plates were overlaid with LB soft agar (0.8% agar) inoculated with *Chr. violaceum* WT (OD_600_~0.1), or with *Chr. violaceum* CV026 (OD_600_~0.1) supplemented with 5 µM of 3-Oxo-C6-HSL. Once the plates solidified, 9 mm diameter wells were punched and filled with 100 µL of the cell-free supernatant of each FI. As the cell-free supernatants of all fish isolates tested do not interfere with the biosensor’s growth, the zones of violacein pigmentation inhibition around the wells after 48 h at 30 °C were considered a positive result for QQ extracellular activity. The laboratory strain *B. subtilis* 168 was used as a control for FIs bacterial growth and strains FI314, FI330, and FI442 were used as a positive control for QQ activity, as described earlier [27]. All digital photos were taken with a Sony IMX240 camera and zones (in mm) of pigment inhibition recorded.

### 4.3. AHLs Enzymatic Inactivation by Isolates’ Extracellular Compounds 

Fish isolates’ cell-free supernatant was tested for AHL enzymatic degradation of 30 µM of 3-Oxo-C6-HSL, using a well-diffusion method, as follows: LB plates were overlaid with 8 mL of LB soft agar (0.8% agar) previously inoculated with *Chr. violaceum* CV026 (OD600 ~ 0.1). After plate solidification, 9 mm diameter wells were punched and filled with 100 µL of AHL-SUP reaction mixtures, (cell-free supernatant of each FI strain mixed with 30 µM of 3-Oxo-C6-HSL. To evaluate enzymatic activity, the AHL-SUP reaction mixtures were incubated at room temperature, with agitation at 120 rpm, for 0 h and 24 h, before using in the bioassays with *Chr. violaceum* CV026. LB media with and without the same concentration of 3-Oxo-C6-HSL were used as negative controls. An intensity and size reduction in the violacein pigment halo on the biosensor submitted to both reaction mixtures 0 h and 24 h was considered a positive result for enzymatic degradation.

The method described by Edwin A. Yates [48] was used to elucidate whether the enzymatic degradation of the 3-Oxo-C6-HSL could be due to a lactonase. Reaction mixtures were acidified with 10 N HCL to pH 2.0, and then used again in a new bioassay with *Chr. violaceum* CV026, as described. Since lactonase enzymatic reaction is pH-mediated and reversible by acidification, the restoration of the violacein pigment halo on *Chr. violaceum* CV026 is considered positive for lactonase activity. All digital photos were taken with a Sony IMX240 camera and zones (in mm) of pigment inhibition were recorded.

### 4.4. Design of QQ Primers

To obtain a set of primers specific to genes encoding putative QQ enzymes, an initial search was conducted at the Protein Knowledgebase – UniProtKB for “Bacillus Quorum Quenching lactonase”. Selected enzymes included YtnP (probable QQ lactonase) (https://www.uniprot.org/uniprot/O34760, accessed on 9 June 2019) and AiiA (N-acyl homoserine lactonase) (https://www.uniprot.org/uniprot/Q9L8R8, accessed on 9 June 2019). The protein sequence of each enzyme was used to search for similar proteins in the translated nucleotide database using NCBI (https://www.ncbi.nlm.nih.gov, accessed on 9 June 2019). Nucleotide sequence alignments with ClustalW (GenomeNet, Kyoto University, Japan) allowed for the detection of regions of sequence conservation, used to design a pair of primers for each enzyme-encoding gene (*ytnP* and *aiiA*) with the SnapGene software version 5.2.3 (GSL Biotech LLC, San Diego, CA, USA) (Table 1).

### 4.5. PCR Amplification of 16S rRNA Genes and Genes Coding for Putative QQ Enzymes 

Fish isolates’ genomic DNA (gDNA) was extracted using ZymoBIOMICS DNA Miniprep kit (Zymoresearch, Irvine, CA, USA). For strain identification, a DNA fragment containing an almost complete sequence of 16S rRNA gene (~1465 bp) was amplified using primers 16S-27F and 16S-1492R (Table 1). Similarly, putative QQ-genes were amplified using primers ytnP-149F and ytnP-708R and, aiiA-1F and aiiA-584R (Table 1). A putative *aiiA* gene was also amplified using a set of primers described by Kalia, Raju and Purohit [37] for Firmicutes (267 bp) (Table 2).

Each Polymerase Chain Reaction (PCR) contained 5×MyTaq Reaction Buffer (Bioline, London, United Kingdom), 10 μM of each primer (STAB Vida, Lisboa, Portugal), 1 U of Mytaq DNA polymerase enzyme (Bioline, United Kingdom), and DNA template. The program consisted of an initial denaturation step at 95 °C for 60 s, followed by 35 cycles of denaturation at 95 °C for 30 s, annealing at 52 °C (for 16S rRNA), 49 °C (for *ytnP*), 45 °C (for *aiiA*) or 50 °C (for *aiiA* using Kalia, Raju and Purohit [37] primers) for 30 s and, a final extension at 72 °C for 10 s. All PCR reactions were performed using a T100^TM^ Thermal Cycler (Bio-Rad, Algés, Portugal). 

Phylogenetic analysis was performed with the GenBank non-redundant nucleotide database (Blastn) and the GenBank protein database using a translated nucleotide sequence (Blastx) with BLAST (http://www.ncbi.nlm.nih.gov, accessed on 15 January 2020). The amino acid sequences of the putative AHL-lactonase enzymes were aligned with ClustalW software (https://www.genome.jp/tools-bin/clustalw, accessed on 15 January 2020) and the phylogenetic tree was built using the neighbor-joining method available in ClustalW software.

### 4.6. AHL Production Profile of Fish Pathogens

AHLs production by fish pathogens was explored using a cross-feeding method, in which *Aeromonas bivalvium*, *A. hydrophila* LMG 2844, *A. salmonicida* LMG 3780, *A. veronii*, *Edwardsiella tarda* LMG 2793, *Photobacterium damselae* subsp. *piscicida*, *Ph. damselae* subsp. *damselae* LMG 7892, *Shigella sonnei* LMG 10473, 2, *Vibrio anguillarum* DSM 21597, *V. harveyi*, *V. parahaemolyticus* LMG 2850 and *V. vulnificus* LMG 13545 were cross-fed alongside (1 cm apart) the biosensor *Chr. violaceum* CV026 on an LB plate. Plates were incubated for 48 h at 30 °C and the induction of violacein production on the biosensor was considered a positive result for AHLs production by the tested fish pathogens. 

Pathogens’ AHLs-production kinetics were evaluated in the supernatant of *A. salmonicida*, *A. veronii*, *A. bivalvium* and *E. tarda*. In brief, an inoculum (OD_600_~0.05) of *A. bivalvium*, *A. salmonicida*, *A. veronii* and *E. tarda* was prepared in BHI medium and grown for 48 h at 25 °C (or 37 °C in the case of *E. tarda*), 140 rpm. Every 2 hours (or every 4 hours after the first 24 h of growth), the optical density (OD_600_) was measured and the pathogens’ supernatant was obtained by centrifugation for 10 min at 16,000× *g*, and filtration with 0.22 µm cellulose acetate filter. The revelation of AHLs in the pathogen’s supernatant was performed by overlaying a LB agar plate with 8 mL of LB soft agar (0.8% agar) inoculated with *Chr. violaceum* CV026 (OD_600_~0.1). After plate solidification, 9 mm diameter wells were punched and filled with 100 µL of each pathogen’s supernatant. Plates were incubated for 48 h at 30 °C and violacein pigment halos around the wells were considered a positive result. All digital photos were taken with a Sony IMX240 camera (Sony, Tokyo, Japan)and zones (in mm) of recorded pigment inhibition.

### 4.7. Extraction of Fish Pathogens’ AHLs 

The AHLs produced by *A. salmonicida*, *A. veronii*, *A. bivalvium*, and *E. tarda* were extracted as described in [78], with some modifications. In brief, an inoculum of each bacterial strain (OD_600_~0.05) was prepared in 25 mL of BHI medium from overnight cultures. *A. salmonicida*, *A. veronii*, *A. bivalvium* were grown for 6 h at 25 °C, and *E. tarda* was grown for 14 h at 37 °C, 140 rpm. Pathogens’ supernatant was obtained from centrifugation for 10 min at 16,000× *g*, and filtration with 0.22 µm cellulose acetate filter and mixed with an equal volume of acidified ethyl acetate (0.1% of acetic acid). Mixtures were shaken for 30 min, followed by phase separation. The organic phases were pooled and stored at 4 °C. The extraction procedure was repeated three times to improve AHLs extraction. The pooled fraction was concentrated in a rotary evaporator at room temperature. Finally, the dried extract was dissolved in 500 μL of ethyl acetate and stored at 4 °C until use.

### 4.8. Fish Isolates QQ Activity on Fish Pathogens’ AHLs 

Fish isolates’ QQ activity on *A. salmonicida*, *A. veronii*, *A. bivalvium*, and *E. tarda* AHL’s was evaluated by overlaying LB plates with 8 mL of LB soft agar inoculated with *Chr. violaceum* CV026 (OD_600_~0.05) supplemented the natural AHLs extracted from the fish pathogens (10, 20, 40, 60, 80 and 100 μL). Once the plates solidified, 9 mm diameter wells were punched and filled with 100 µL of cell-free supernatant of each fish isolate strain. As in previous bioassays, zones of violacein inhibition around the wells (without interference on biosensor growth) after 48 h at 30 °C were considered a positive result for QQ activity. All digital photos were taken with a Sony IMX240 camera and zones (in mm) of pigment inhibition were recorded.

### 4.9. Ethics Statement

Zebrafish experiments and handling were approved by the Animal Welfare Committee of the Interdisciplinary Centre of Marine and Environmental Research (CIIMAR), performed by trained scientists (with FELASA category C), carried out in a registered installation (N16091.UDER), in compliance with the European directive 2010/63/EU for the care and use of laboratory animals.

### 4.10. Zebrafish Larvae General Care 

Zebrafish embryos were obtained from a wild-type zebrafish broodstock, and incubated in egg water at 28 °C under a photoperiod of 14 h of light:10 h of darkness until hatching. After hatching, larvae were kept in the same conditions and from 6 dpf were fed twice a day (diet containing 36.7% of total crude protein and 15% of total lipids). After each experiment, the surviving larvae were euthanized with a lethal dose of tricaine methanesulfonate (MS-222, 300 mg L^−1^).

### 4.11. Isolates Extracts Preparation and Testing for Toxicity in Zebrafish Larvae 

The extracts of the 3 most promising QQ fish isolates were obtained by freeze-drying the filtered cell-free supernatant of 8 h cultures and resuspending it in sterile 1×PBS. 

The evaluation of their in vivo toxicity was performed using zebrafish larvae (*Danio rerio*) as a model, following the Organisation for Economic Co-operation and Development (OECD) Guidelines for Fish Embryo Toxicity Tests [79]. 

Zebrafish larvae at 4 days post-fertilization (dpf) were distributed into 6-well plates containing 10 larvae/well in 5 mL of egg water (26.4 mg L^−1^ of Instant Ocean® Salt) and exposed to fish isolates’ extracts with concentrations ranging from 67.5 μg mL^−1^ to 1 mg mL^−1^. Larval mortality was recorded at 4, 5, 6, and 7 dpf, and the dead larvae were removed and discarded. Larvae kept in egg water (without treatment) were used as a negative control. The experiment was performed in triplicate to determine the maximum non-toxic extract concentration (MNTC) to be used in subsequent assays. 

### 4.12. E. tarda Infection Model

Zebrafish larvae were used to establish an infection model of *E. tarda* by bath immersion. *E. tarda* was cultured for 24 h in BHI at 37 °C with 140 rpm, pelleted by centrifugation (6000× *g*) at room temperature, washed twice with sterile 1×PBS and then diluted to the correct concentration in 1×PBS. Before the establishment of the infection model, bacterial cell densities ranging from 10^4^ to 10^9^ were tested to evaluate their virulence in zebrafish larvae during 24 h and determine the lowest lethal concentration causing 100% mortalities (5 × 10^8^ CFUs mL^−1^) and the non-lethal dose (1 × 10^7^ CFUs mL^−1^) (data not shown). 

Zebrafish larvae at 10 dpf were distributed into 6-well plates containing 10 larvae/well in 5 mL of Egg water and inoculated with 5 × 10^7^, 1 × 10^8^, and 3 × 10^8^ CFUs mL^−1^ of *E. tarda*. After inoculation, larvae were fed, and the plate was incubated at 28 °C. The *E. tarda* inoculum was kept in the water throughout the whole experiment (24 h). Cumulative mortalities were registered for 24 h and dead larvae found during the assay were removed and discarded. Control groups were included: (i) non-inoculated larvae, with egg-water only; (ii) larvae inoculated with 1×PBS. The experiment was independently performed 3 times.

### 4.13. Fish Isolates’ Protection Assay against E. tarda Infection in Zebrafish Larvae

The fish isolates’ protection of zebrafish larvae against *E. tarda* infection was performed by testing the lyophilized extracts at a final concentration of 250 μg mL^−1^. Before the assay, a pre-treatment experiment was performed, where zebrafish larvae were treated with the extracts once, twice, or thrice for 2 or 24 h before challenge with *E. tarda*, allowing for the establishment of the best protection method. Thus, at 4 dpf, 10 healthy larvae were distributed into each well of a 6-well plate, containing 5 mL of Egg water. Larvae were treated with the extracts once after mouth’s opening (7 dpf), for 2 h at 28 °C and then transferred to new 5 mL egg water. The treated 10 dpf larvae were challenged by immersion with *E. tarda* at 1 × 10^8^ CFU mL^−1^. After inoculation, larvae were fed, and the plate was incubated at 28 °C for 24 h. Cumulative mortalities were registered between 16 and 24 h and the dead larvae were removed and discarded. Control groups were included: (i) non-treated larvae inoculated with *E. tarda*; (ii) non-inoculated larvae; (iii) larvae inoculated with 1×PBS. The experiment was independently performed 3 times.

### 4.14. Statistical Analysis

Survival data were analysed using Kaplan–Meier, and group differences were analysed by the log-rank, using the GraphPad Prism 9 software.

The one-way ANOVA was performed to compare between treatments and the control, using the SPSS 26.0 software (IBM Corp., New York, NY, USA) package for Windows. When *p*-values were significant (*p* < 0.05), means were compared with Dunnett’s test.

## Figures and Tables

**Table 1:** Oligonucleotide primers used in this study.

Name	Sequence (5′-3′)	Amplicon (bp)	Reference
**16S rRNA**			
27F	AGAGTTTGATCMTGGCTCAG	1465	[38]
1492R	GGYTTACCTTGTTAYGACTT		[38]
**N-acyl homoserine lactonase/*aiiA* ^a^**			
aiiA–309F	TCACTTACATTTTGATCATGCAGGAGGAAA	267	[37]
aiiA–576R	TCCGGTTCAGTTTTATTAACGATTGATGCA		[37]
aiiA–1F	ATGACAGTAAAGAAGCTTTATT	584	This study
aiiA–584R	CATCTTCAAAATTCTCTTTCG		This study
**Probable quorum-quenching lactonase/*ytnP* ^b^**			
ytnP–149F	ATCGGATAATCATCGTAAGC	559	This study
ytnP–708R	ATTGAACTAAGAACAGACCC		This study

^a^ gene name in *B. subtilis* strain BS2 genome, whose sequence was used to design the oligonucleotide primers for *aiiA*. ^b^ gene name in *B. subtilis* strain 168 genome, whose sequence was used to design the oligonucleotide primers for *ytnP*.

**Table 2:** Identification of putative QQ-related genes amplified from gDNA of fish isolates (FI).

Gene	FI nº	Closest known Protein ^a^	QC (%) ^b^	Identity (%) ^c^
*ytnP*	314	ytnP-like metallo-hydrolase	99	100
	436	MBL fold metallo-hydrolase	100	100
	464	DAK2 domain-containing protein	88	99.2
*aiiA*	314	MBL fold metallo-hydrolase – Rhodanese Homology Domain	96	100
	383	MBL fold metallo-hydrolase – Rhodanese Homology Domain	95	100

^a^ Closest known protein using BLASTx based on partial sequences of QQ genes (~300–400 nt). ^b^ Query Cover—the percentage of the query sequence covered by the reference sequence. ^c^ Percent Identity—the percentage of similarity between the query sequence and the reference sequence.

**Table 3:** Bacterial strains used in this study.

Bacterial Species	Strain	Origin/Source ^a^
Fish pathogens		
*Aeromonas salmonicida*	LMG 3780	BCCM/LMG
*Aeromonas veronii*	Fish isolate	NUTRIMU collection
*Aeromonas bivalvium*	Fish isolate	NUTRIMU collection
*Aeromonas hydrophila* subsp. *hydrophila*	LMG 2844	BCCM/LMG
*Vibrio anguillarum*	DSM 21597	DSMZ
*Vibrio harveyi*	Fish isolate	NUTRIMU collection
*Vibrio parahaemolyticus*	LMG 2850	BCCM/LMG
*Vibrio vulnificus*	LMG 13545	BCCM/LMG
*Photobacterium damselae* subsp. *damselae*	LMG 7892	BCCM/LMG
*Photobacterium damselae* subsp. *piscicida*	*Lg_h41/01_*	[39]
*Tenacibaculum maritimum*	LMG 11612	BCCM/LMG
*Edwarsiella tarda*	LMG 2793	BCCM/LMG
*Shigella sonnei*	LMG 10473	BCCM/LMG
*Bacillus subtilis* subsp. *subtilis*	168 (BGSC1A1)	A.O. Henriques
*Chromobacterium violaceum* WT	CECT 494	CECT
*Chromobacterium violaceum* CV026	CECT 5999	CECT

^a^ Bacterial strains were obtained from bacterial collections (BCCM/LMG, Belgian Coordinated Collections of Microorganisms, Laboratory of Microbiology, Department of Biochemistry and Microbiology, Faculty of Sciences of Ghent University, Ghent, Belgium; DSMZ, DSM Collection, German Collection of Microorganisms and Cell Cultures, Braunschweig, Germany; CECT, Spanish Type Culture Collection, Valencia, Spain), from our laboratory stocks (NUTRIMU collection) or kindly supplied by M.A. Morinigo (Universidad Málaga), and A. O. Henriques (Instituto de Tecnologia Química e Biológica António Xavier, Universidade Nova de Lisboa, Portugal).

## Data Availability

16S rRNA gene sequences of fish isolates described in this manuscript have been deposited in GenBank with the accession numbers provided in Table 2. Authors confirm that all relevant data are included in the article.

## Supplementary Materials

The following are available online at https://www.mdpi.com/article/10.3390/md19110602/s1, Table S1: Identification of the 10 fish-gut isolates with QQ activity, using 16S rRNA gene analysis. Figure S1: Amino acid sequence comparison of the putative AHL-lactonases with known AHL-lactonase enzymes. Figure S2: Detection of AHLs production by gram-negative fish pathogens using *Chr. violaceum* CV026 as a biosensor. Figure S3: Growth curves and QQ kinetics of FI314, FI436 and FI464. Figure S4: Toxicity of fish isolates’ extracellular compounds and establishment of *E. tarda* infection model in zebrafish larvae. Figure S5: Flow diagram of the methodology used for evaluating FI isolates QQ activity.
