# Supplementary material for: Bacillus spp. Inhibit Edwardsiella tarda Quorum-Sensing and Fish Infection
Source: Mar Drugs. 2021 Oct 23;19(11):602. doi: 10.3390/md19110602 (PMC8623655; doi:10.3390/md19110602)
Supplement: Supplementary file 1 [file marinedrugs-19-00602-s001.zip › marinedrugs-1402283-supplementary/Santos.et.al_Supplementary Figures.pdf]

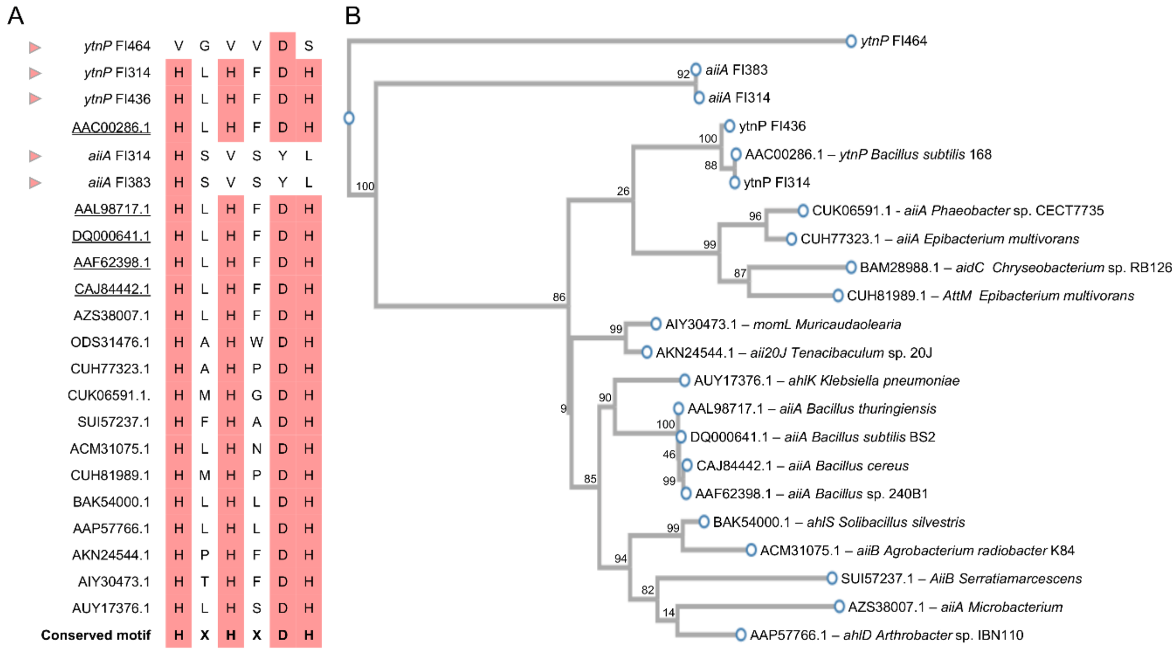

Figure S1. Amino acid sequence's comparison of the putative AHL-lactonases with known AHL-lactonase enzymes. **(A)** Amino acid sequences corresponding to putative AHL-lactonases from *aiiA* (*aiiA* FI314 and *aiiA* FI383) and *ytnP* (*ytnP* FI314, *ytnP* FI436 and *ytnP* FI464) were aligned with known AHL-lactonases enzymes from MBL fold metallohydrolase family, and the conserved motif "HXHXDH" is represented in red. Columns with the same colour represent the amino acid or motif conservation between the sequences. In underlined and double underlined lettering are the known *aiiA* and *ytnP* enzymes from *Bacillus* species, respectively. **(B)** Phylogenetic analysis based on the amino acid sequences of the putative AHL-lactonases (*aiiA* FI314, *aiiA* FI383, *ytnP* FI314 and *ytnP* FI464) and known homologous AHL-lactonases enzymes from different species including *B. subtilis*, *B. thuringiensis*, *B. cereus*, *Microbacterium*, *Epibacterium multivorans*, *Phaeobacter* sp., *Chryseobacterium* sp., *Serratia marcescens*, *Arthrobacter* sp., *Tenacibaculum* sp., *Muricaudaolearia*, *Klebsiella pneumoniae*, *Agrobacterium radiobacter* and *Solibacillus silvestris*. Phylogenetic analysis and dendrogram construction were performed using ClustalW software.

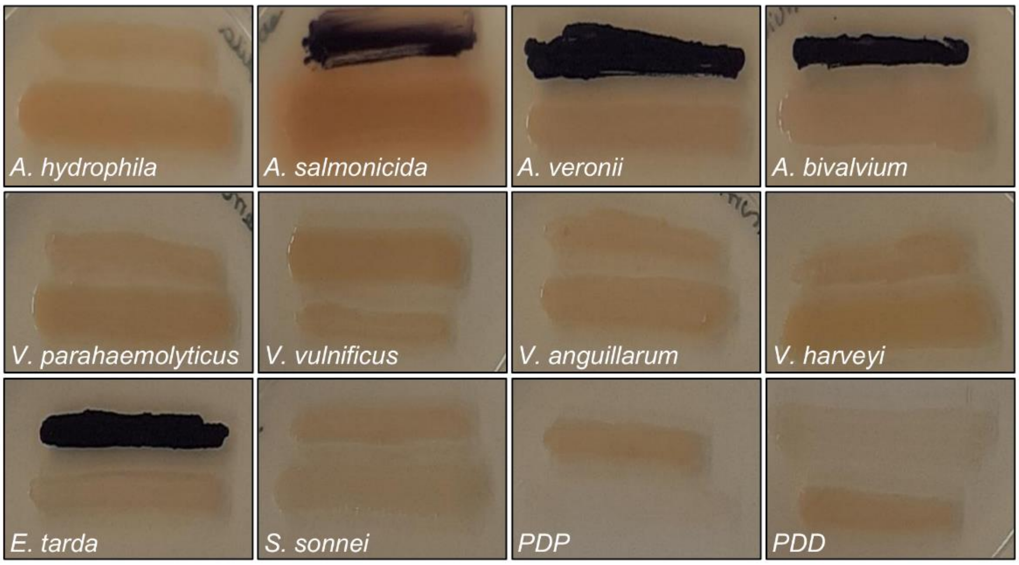

Figure S2. Detection of AHLs production by gram-negative fish pathogens using *Chr. violaceum* CV026 as a biosensor. *Aeromonas bivalvium*, *A. hydrophila*, *A. salmonicida*, *A. veronii*, *Edwardsiella tarda*, *Photobacterium damselae* subsp. *piscicida* (PDP), *Ph. damselae* subsp. *damselae* (PDD), *Shigella sonnei*, *Tenacibaculum maritimum*, *Vibrio anguillarum*, *V. harveyi*, *V. parahaemolyticus* and *V. vulnificus* were streaked in parallel (1 cm apart) to the biosensor on LB agar plates. Induction of violacein pigment production by the biosensor was considered a positive result for AHLs production. All photos were taken with a Sony IMX240 camera and are at the same scale.

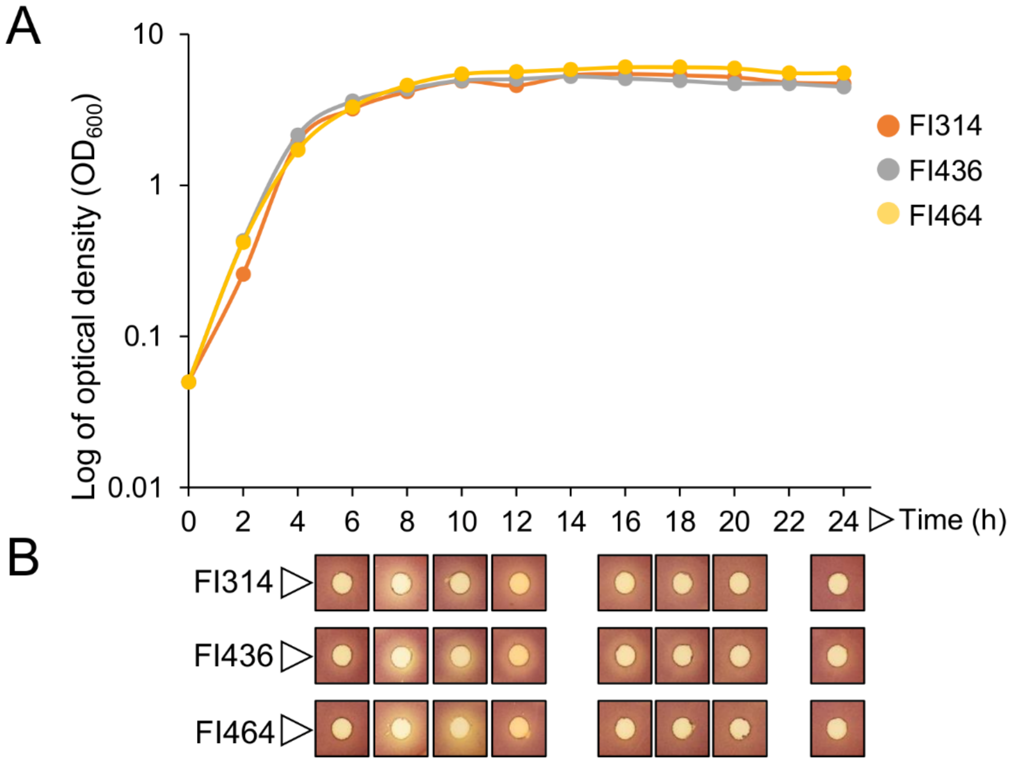

Figure S3. Growth curves and QQ kinetics of FI314, FI436 and FI464. **(A)** Optical density of FI isolates grown in LB medium at 37 °C, 140 rpm. **(B)** *Chr. violaceum* CV026 biosensor violacein pigment production inhibition around the wells containing the cell-free supernatant of sporeforming fish isolates (FI) at different time points (6, 8, 10, 12, 16, 18, 20, 24H). All photos were taken with a Sony IMX240 camera and are at the same scale.

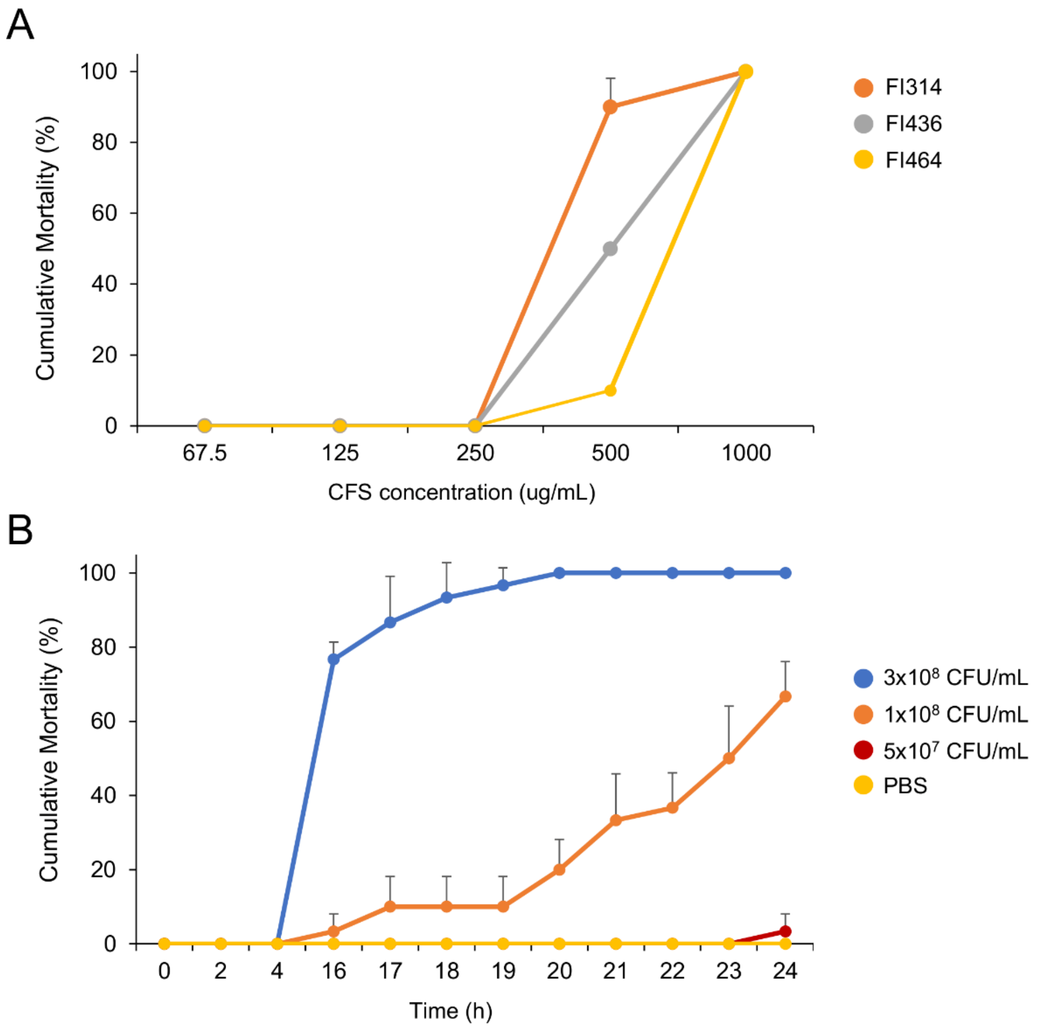

Figure S4. Toxicity of fish isolates (FI) extracellular compounds and establishment of *E. tarda* infection model in zebrafish larvae. **(A)** Toxicity of zebrafish larvae when exposed to several concentrations (67.5, 125, 250, 500 and 1000 µg mL^{-1}) of the FI cell free supernatant extracts. Mortalities were recorded each 24h for 3 days. Data is composed by three independent experiments using 10 larvae per concentration in each experiment. **(B)** Evolution of cumulative mortality (for 24h) of zebrafish larvae infected with *E. tarda* at three concentrations ($5x10^7$, $1x10^8$ and $3x10^8$ CFUs mL^{-1}). Data is composed by three independent experiments using 10 larvae per treatment in each experiment.

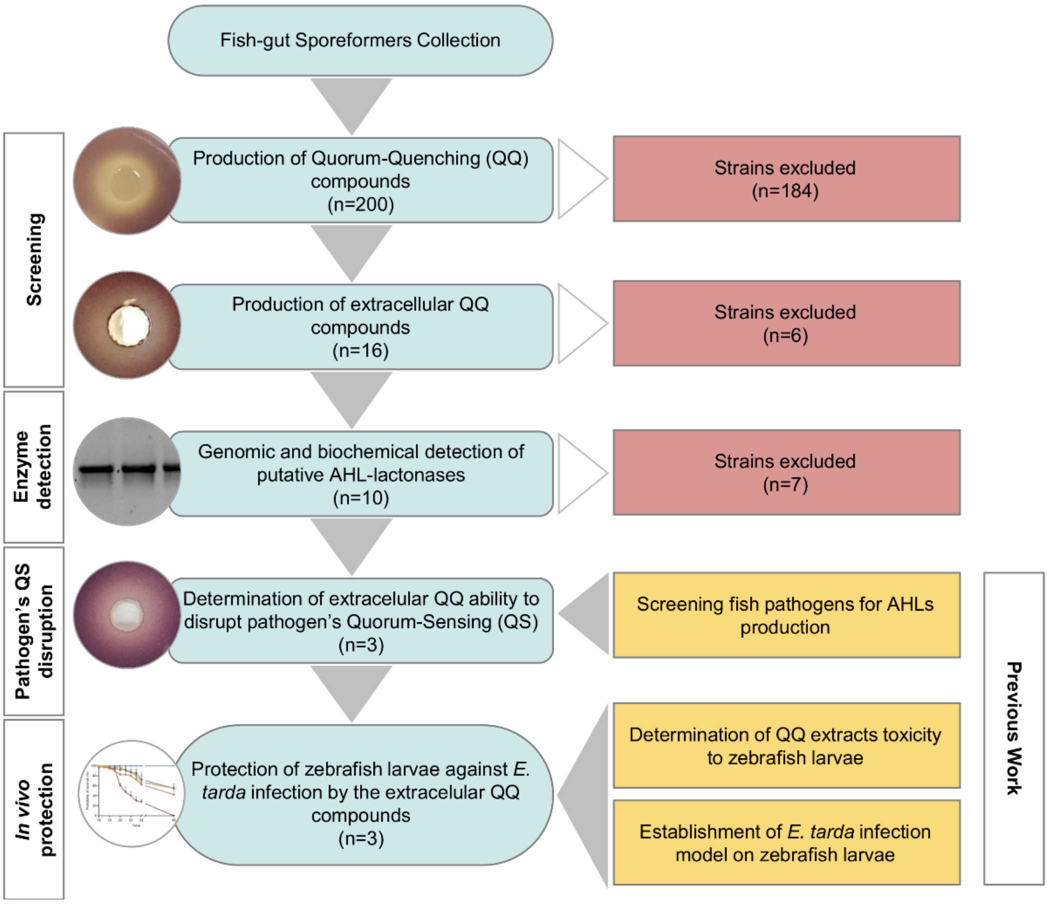

Figure S5. Flow diagram of the methodology used for evaluating FI isolates QQ activity
