# Supplementary material for: Bacillus spp. Inhibit Edwardsiella tarda Quorum-Sensing and Fish Infection
Source: Mar Drugs. 2021 Oct 23;19(11):602. doi: 10.3390/md19110602 (PMC8623655; doi:10.3390/md19110602)
Supplement: Supplementary file 1 [file marinedrugs-19-00602-s001.zip › marinedrugs-1402283-supplementary/Santos.et.al_Supplementary Tables.pdf]

Supplementary Table S1. Identification of the 10 fish-gut isolates with QQ activity, using 16S rRNA gene analysis.

Isolate nº	Closest known species[a]	% Q.Cover[b]	% Identity[c]	GenBank accession
FI314	*Bacillus subtilis*	100	99.9	MT081448
FI330	*Bacillus subtilis*	100	100	MT081452
FI333	*Bacillus amyloliquefaciens*	100	99.7	MT081453
FI335	*Bacillus velezensis*	100	100	MT081454
FI346	*Bacillus subtilis*	100	99.7	OK094928
FI383	*Bacillus velezensis*	99	99.4	OK094929
FI423	*Bacillus amyloliquefaciens*	100	99.7	MT081476
FI436	*Bacillus velezensis*	100	99.4	MT081479
FI442	*Bacillus subtilis*	100	100	MT081480
FI464	*Bacillus pumilus*	99	98.9	MT081483

[a] Closest known species using BLASTn based on partial sequences of 16S rRNA gene (~1000nt)
[b] Query Cover - the percentage of the query sequence covered by the reference sequence
[c] Percent Identity - the percentage of similarity between the query sequence and the reference sequence
